# Supplementary material for: Factors Associated with the Acceptance of New Technologies for Ageing in Place by People over 64 Years of Age
Source: Int J Environ Res Public Health. 2022 Mar 3;19(5):2947. doi: 10.3390/ijerph19052947 (PMC8910177; doi:10.3390/ijerph19052947)
Supplement: Supplementary file 1 [file ijerph-19-02947-s001.zip › ijerph-1608163-Table S1.pdf]

Modificado de TAM (Modelo de Aceptación Tecnológica)

Teniendo en cuenta un dispositivo tecnológico para el envejecimiento en el hogar (por ejemplo un asistente de voz), responde a las siguientes preguntas de 1 (menos o nada de acuerdo) a 5 (totalmente de acuerdo)

**Facilidad de uso percibida**
01 Siento que podría usar bien este sistema
1
2
3
4
5
NS/NC
02 Creo que me sería fácil aprender a utilizarlo
1
2
3
4
5
NS/NC
03 Puede que, por mi desconocimiento, no sea capaz de usarlo (valores invertidos)
1
2
3
4
5
NS/NC
**Utilidad percibida**
04 Estoy entusiasmado(a) con la idea de utilizarlo
1
2
3
4
5
NS/NC
05 Siento que usando algo similar, podría sentirme menos solo(a)
1
2
3
4
5
NS/NC
**Actitud hacia el uso**

06 ¿Cuántas experiencia tiene en el uso de dispositivos?
1
2
3
4
5
NS/NC
07 Creo que para utilizar un dispositivo necesitaré ayuda y será difícil para mí. (valores invertidos)
1
2
3
4
5
NS/NC
08 Creo que el dispositivo me ayudará bastante y hará lo que yo digo
1
2
3
4
5
NS/NC
**Relevancia**
09 Tengo claro para que sirve un dispositivo
1
2
3
4
5
NS/NC
10 Interactuar con el dispositivo no parece difícil
1
2
3
4
5
NS/NC
11 Cuánto tiempo al día usaría el sistema
1
2
3
4
5

NS/NC

Modified from TAM (Technology Acceptance Model)

Taking into account a technological device for aging in place (for example a voice assistant), answer the following questions from 1 (less or not at all) to 5 (totally agree)

**Perceived ease of use**
01 I feel that I could use this system well
1
2
3
4
5
NS/NC
02 I think it would be easy for me to learn how to use it
1
2
3
4
5
NS/NC
03 I may not be able to use it due to my ignorance (inverted values)
1
2
3
4
5
NS/NC
**perceived utility**
04 I am excited about the idea of using it
1
2
3
4
5
NS/NC
05 I feel that by wearing something similar, I could feel less alone
1
2
3
4
5
NS/NC
**attitude towards use**

06 How much experience do you have in using devices?
1
2
3
4
5
NS/NC
07 I think that to use a device I will need help and it will be difficult for me. (reversed values)
1
2
3
4
5
NS/NC
08 I think the device will help me a lot and will do what I say
1
2
3
4
5
NS/NC
**Relevance**
09 I am clear about what a device is for
1
2
3
4
5
NS/NC
10 Interacting with the device does not seem difficult
1
2
3
4
5
NS/NC
11 How much time per day would you use the system
1
2
3
4
5

NS/NC
